# External root resorption and rapid maxillary expansion in the short-term: a CBCT comparative study between tooth-borne and bone-borne appliances, using 3D imaging digital technology

**DOI:** 10.1186/s12903-023-03280-9

**Published:** 2023-08-12

**Authors:** Rosalia Leonardi, Vincenzo Ronsivalle, Gaetano Isola, Marco Cicciù, Manuel Lagravère, Carlos Flores-Mir, Antonino Lo Giudice

**Affiliations:** 1 Department of General Surgery and Medical-Surgical Specialties, Section of Orthodontics, University of Catania, Catania, Italy; 2 Department of General Surgery and Medical-Surgical Specialties, Section of Oral Surgery, University of Catania, Catania, Italy; 3 Department of General Surgery and Medical-Surgical Specialties, Section of Periodontology, University of Catania, Catania, Italy; 4 Orthodontic Graduate Program, University of Alberta, Edmonton, AB Canada

**Keywords:** RME, Tooth-borne RME, Bone-borne-RME, ERR, Root resorption, Maxillary expansion, Orthodontics

## Abstract

**Background:**

The aim of the study was to analyze and compare external root resorption (ERR) in patients treated with tooth-borne (TB) and bone-borne (BB) rapid maxillary expansion (RME).

**Methods:**

The sample included 40 subjects who received tooth-borne RME (TB group, average age: 13.1 ± 1.08 years) or bone-borne RME (BB group, average age: 14.5 ± 1.11 years) and Cone-beam computed tomography (CBCT) scans before treatment (T0) and after 3-month of retention (T1). A specific 3D Imaging technology was used to generate 3D models of posterior dentition (M1 = maxillary first molars, P2 = second premolars, P1 = first premolar) and calculate volumetric data (mean and percentage values) and shape changes, the latter obtained from deviation analysis between the radicular models at different time points. Evaluation of radicular length changes was performed for each tooth. Data were statistically analysed to perform intra-timing and inter-groups comparisons.

**Results:**

A significant reduction of radicular volume and length was found in posterior dentition in both groups (*p* < 0.05), and the M1 (volume) and its palatal root (length) were mostly involved in this response. No differences were found between M1, P1 and P2 (*p* > 0.05) when volumetric changes were calculated as percentage of the total volume. Deviation analysis revealed that the radicular areas mostly affected by shape change were the apex and bucco-medial side. The amount of ERR was significantly greater in TB group compared to BB group.

**Conclusions:**

BB-RME treatment could reduce the amount of ERR at the post-expansion stage.

## Introduction

Transverse maxillary deficiency is a malocclusion with a prevalence of 8–10% among adolescents or adults [1]. The treatment of this malocclusion demands to increase the transverse widths of the maxilla through the opening of the mid‐palatal suture using a maxillary expander [2]. Rapid maxillary expansion (RME) is the most frequent protocol used to expand the maxilla, and a tooth-borne (TB) expander is the conventional appliance used for this purpose. In TB appliances, heavy forces are transferred to the mid‐palatal suture through anchored teeth [3, 4]. When this force exceeds the resistance of maxillary sutural articulations, the maxillary palatal suture separates, and skeletal expansion begins. Besides the benefits of RME, unwanted dento-alveolar side effects have been documented with tooth-borne expander, including external root resorption [5–7]. In this regard, different anchorage systems, such as tissue-borne and bone-borne expanders, have been recommended to reduce the dental-alveolar side effects produced by RME [7–10].

The development of root resorption after orthodontic treatment has been evaluated through conventional radiographs and light microscope [7, 8], and with three-dimensional methods (3D) such as scanning electron microscope (SEM) [11] and micro-tomography (micro-CT) [9]. Also, cone-beam computed tomography (CBCT) has proven a comparable accuracy to Micro-CT for the assessment of ERR [12]. In this regard, previous CBCT studies [11, 13–15] analyzed ERR following the application of RME and reported volume loss in the maxillary first molars, first premolars and second premolars [11, 13–15].

Furthermore, using engineering software and CBCT images, it is possible to generate 3D anatomical models and analyze the surface changes after treatment by superimposition through a "best-fit" algorithm [16, 17]. Once the surfaces are overlapped, any differences between the 3D superimposed models can be visualized in distinct colors on a 3D color-map, using a surface-to-surface analysis technique. With a analogous 3D technology, Akyalcin [13] found significant changes in the surface area of the posterior dentition immediately after TB-RME, suggesting ERR.

Nevertheless, the literature lacks comparative data on radicular changes after RME with TB and BB expanders. This study aimed to evaluate the changes in radicular volume and length and the surface differences in patients who underwent TB or BB RME by analyzing CBCTs taken before treatment (T0) and after three months of retention (T1). The null hypothesis was that there was no difference in the extent of root resorption between TB and BB at the post-expansion stage.

## Materials and method

The sample of this CBCT study was obtained from previously published materials to avoid unnecessary or additional radiation exposure to the patients. The study was approved by the Health Research Ethics Board of Alberta University–Canada (protocol number: 00075765) and included a sample of adolescents with a diagnosis of transverse skeletal deficiency and who completed the orthodontic treatment at the Orthodontic Clinic of the University of Alberta (Edmonton, Canada, USA). The sample consisted of 40 subjects (17 males, 23 females) with a mean age of 13.8 ± 1.33 years, respectively divided into the TB group (9 males, 11 females; mean age: 13.1 ± 1.08 years) and BB group (8 males, 12 female; mean age: 14.5 ± 1.11). The inclusion criteria were the following: permanent dentition, root completion of M1, P2, and P1, availability of adequate initial and post-retention records (good quality CBCT scans with a large field of view (FOV), photographs, dental casts, and medical history of each patient). The exclusion criteria were: apical lesions and/or root canal treatment of the upper first molars and the first and second premolars, presence of any already diagnosed oral or systemic disease, prescribed medication, previous orthodontic treatment, maxillofacial surgery, or facial trauma. The study has a retrospective design where data retrieved from upper posterior dentition represented the primary dataset and data retrieved from lower dentition served as control dataset.

The characteristics of the RME appliances and the protocol used in this study have been previously described [18]. Briefly, in the TB group, the subjects received a traditional tooth-anchored maxillary expander (hyrax with bands on the first permanent molars and first premolars). The expansion screw was activated twice daily (0.25 mm per turn, 0.5 mm daily). In the BB group, two mini-screws were inserted in the palate between the permanent first molar and the second premolar (length: 12 mm; diameter: 1.5 mm; Straumann GBR System, Andover, MA, USA) and were connected with the expander (Palex II Extra-Mini Expander, Summit Orthodontic Services, Munroe Falls, OH). Activation consisted of 2 turns of the screw (0.25 mm per turn, 0.5 mm daily). Activations were stopped once overexpansion was achieved; afterward, the screw was fixed with light-cured acrylic and kept passively for six months as retention.

Cone beam computed tomography (CBCT) was obtained before RME treatment (T0) and immediately after expansion (T1). Patients were scanned with the same iCAT CBCT Unit (Imaging Sciences International, Hartfield, PA). The setting protocol included a 0.3-mm isotropic voxel, 8.9 s, large field of view at 120 kV and 20 mA. The distance between the two slices was 0.3 mm, which provided accuracy in anatomic registration. All the data sets were acquired and saved using the Digital Imaging and Communications in Medicine (DICOM) format on a personal computer workstation (Lenovo IdeaPad L340 Gaming, Intel Core i7-9750H processor, 15,6″ monitor) for further analysis. Blinding was performed by one investigator who randomly coded DICOM files from the 40 scans. The principal examiner did not have access to the patient’s name and the pre-treatment and post-retention records.

The protocol used in this study for tooth segmentation, model rendering, and deviation analysis was previously validated [16, 17, 19] and consisted of 6 steps.

### Step 1– Generating the segmentation masks and 3D model rendering

**Fig. 1:**
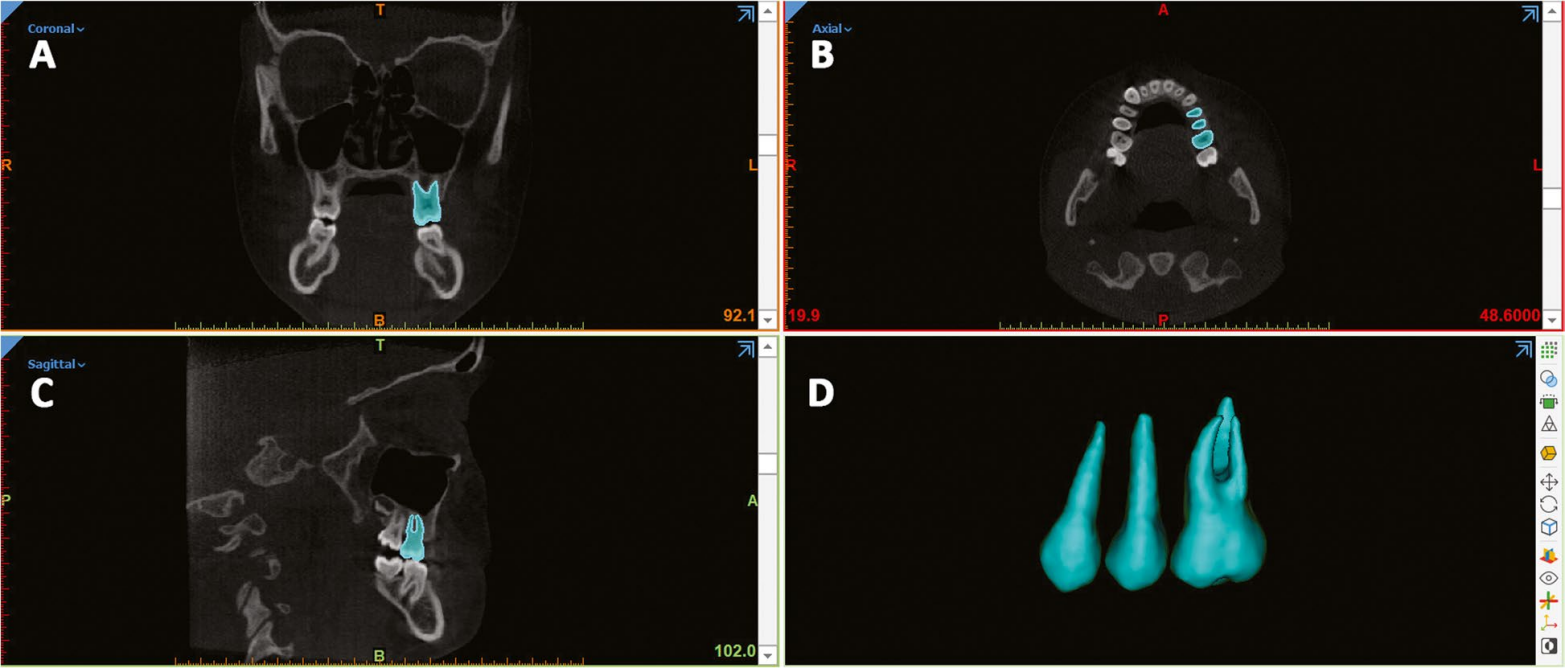
Segmentation masks of P1 (maxillary first premolar), P2 (maxillary second premolar) and M1 (maxillary first molar) using Mimics Medical (Materialise NV vr.21.0, Leuven, Belgium); (**a**-**c**) Coronal, axial and sagittal view, (**d**) 3D rendered tooth model

**Fig. 2:**
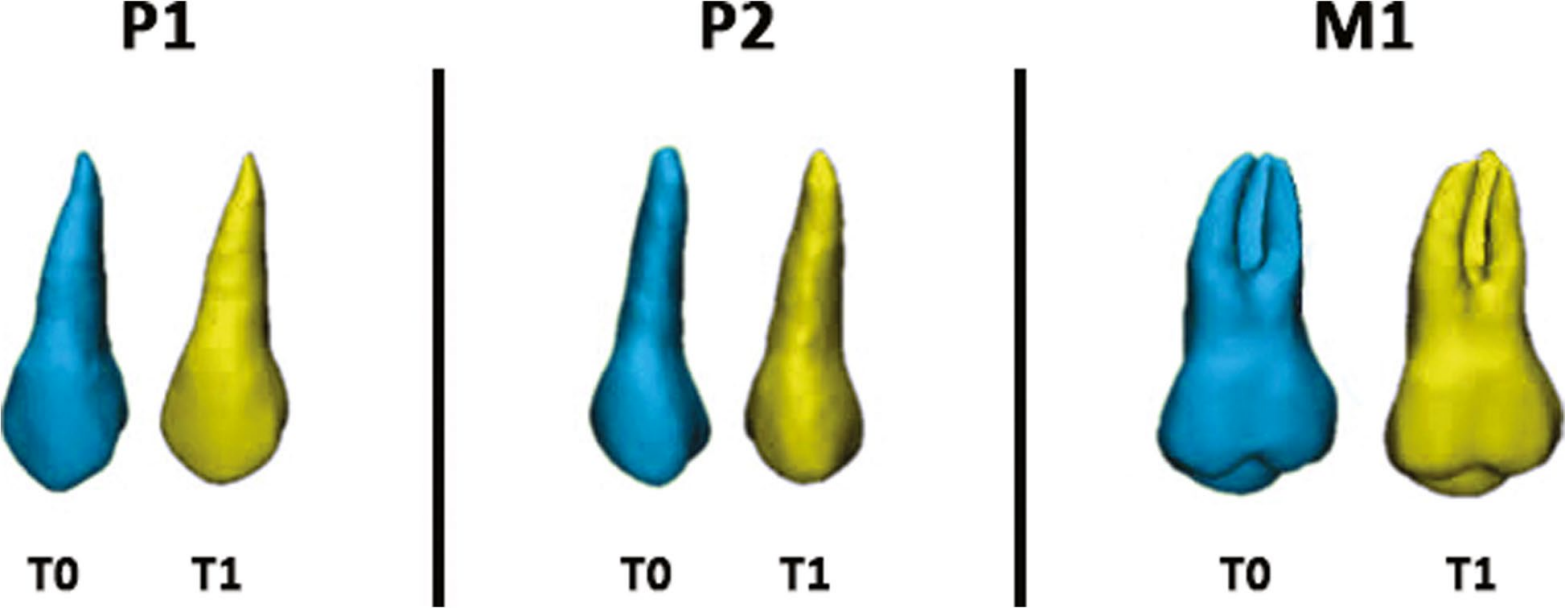
Colour-coded labelling of T0 and T1 tooth models: maxillary first premolar (P1), maxillary second premolar (P2) and maxillary first molar (M1)

Segmentation was done by the same researcher in random order and served to define the anatomic region of interest (ROI) and to create the 3D surface mesh models of posterior dentition. Segmentation masks of first molars (M1), first premolars (P1) and second premolars (P2), both for the right and left side of the upper arch (test sample) and lower arch (control sample), were generated, at each time (T0, T1), with Mimics Medical Software (Materialise NV vr.21.0, Leuven, Belgium) (Fig. 1 a-d). The procedure involved an automatic selection of the threshold value, set to 'teeth', and then adjusted scan by scan. The 3D surface models (.stl file) obtained from the segmentation masks (Fig. 1d) were isolated from the surrounding structures with the "split" function of the software (Fig. 2).

### Step 2 – Root length measurements

**Fig. 3:**
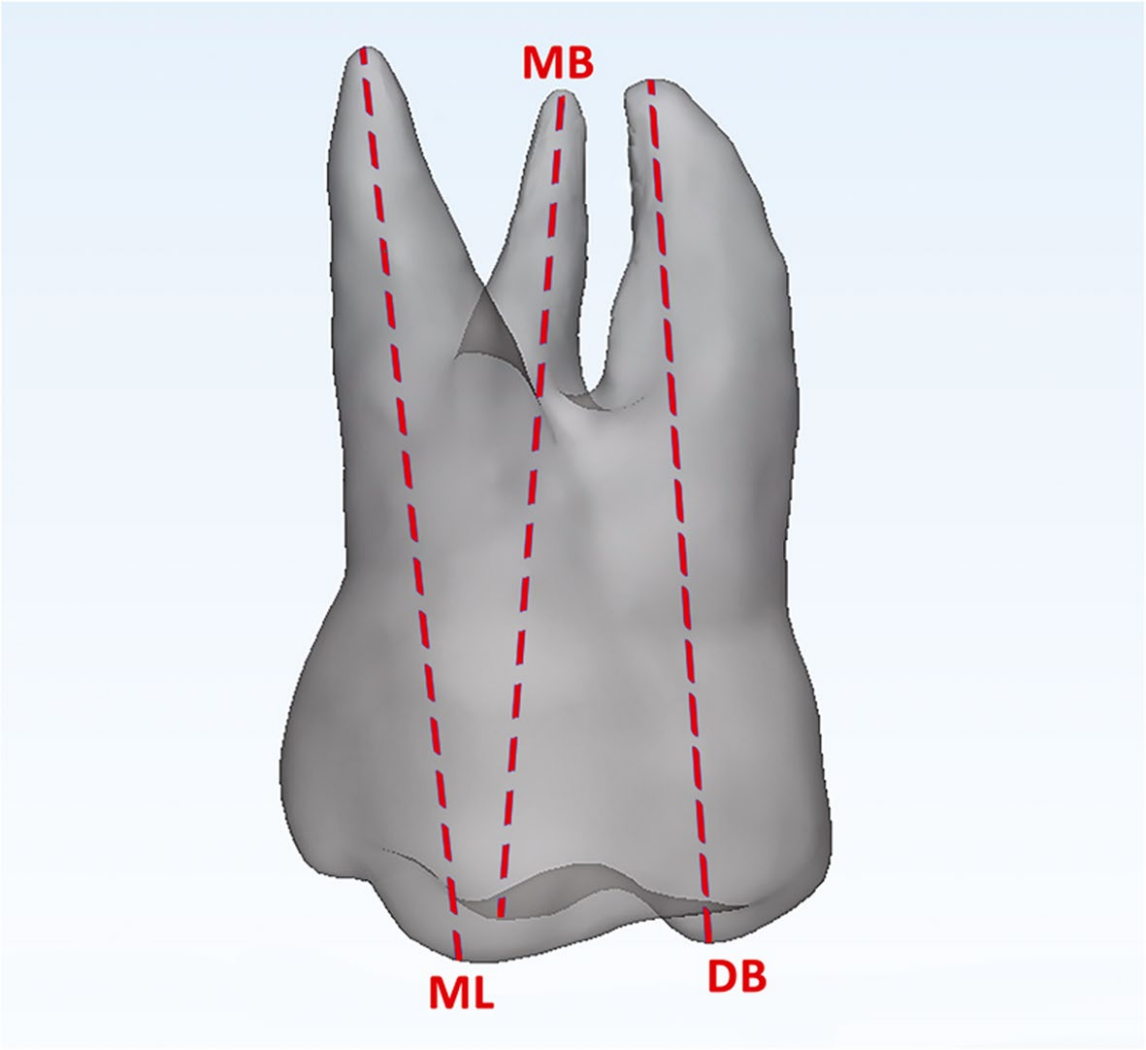
Assessment of root length. Mesiobuccal root assessed as the linear distance between the tip of the mesiobuccal cusp (MB) and the apex of the mesiobuccal root; distobuccal root assessed as the linear distance between the tip of the distobuccal cusp (DB) and the apex of the distobuccal root, and palatal root assessed as the linear distance between the tip of the mesiolingual cusp (ML) and the palatal root apex

The original 3D models of each tooth at T0 and T1were imported onto 3-Matic Medical software (vr. 13.0, Materialise NV, Leuven, Belgium). On the occlusal view, the tip of the mesiobuccal cusp, distobuccal cusp, and mesiolingual cusp of M1 and the tip of the buccal cusp of P1 and P2 were landmarked. Afterwards, the distance between the occlusal tip and the most apical point of the radicular surface was measured for each root (M1m, M1d and M1p measurements) (Fig. 3). If the premolars had two roots, the length from the buccal cusp to the apex of the corresponding root was measured.

### Step 3 – Building a 3D radicular template

**Fig. 4:**
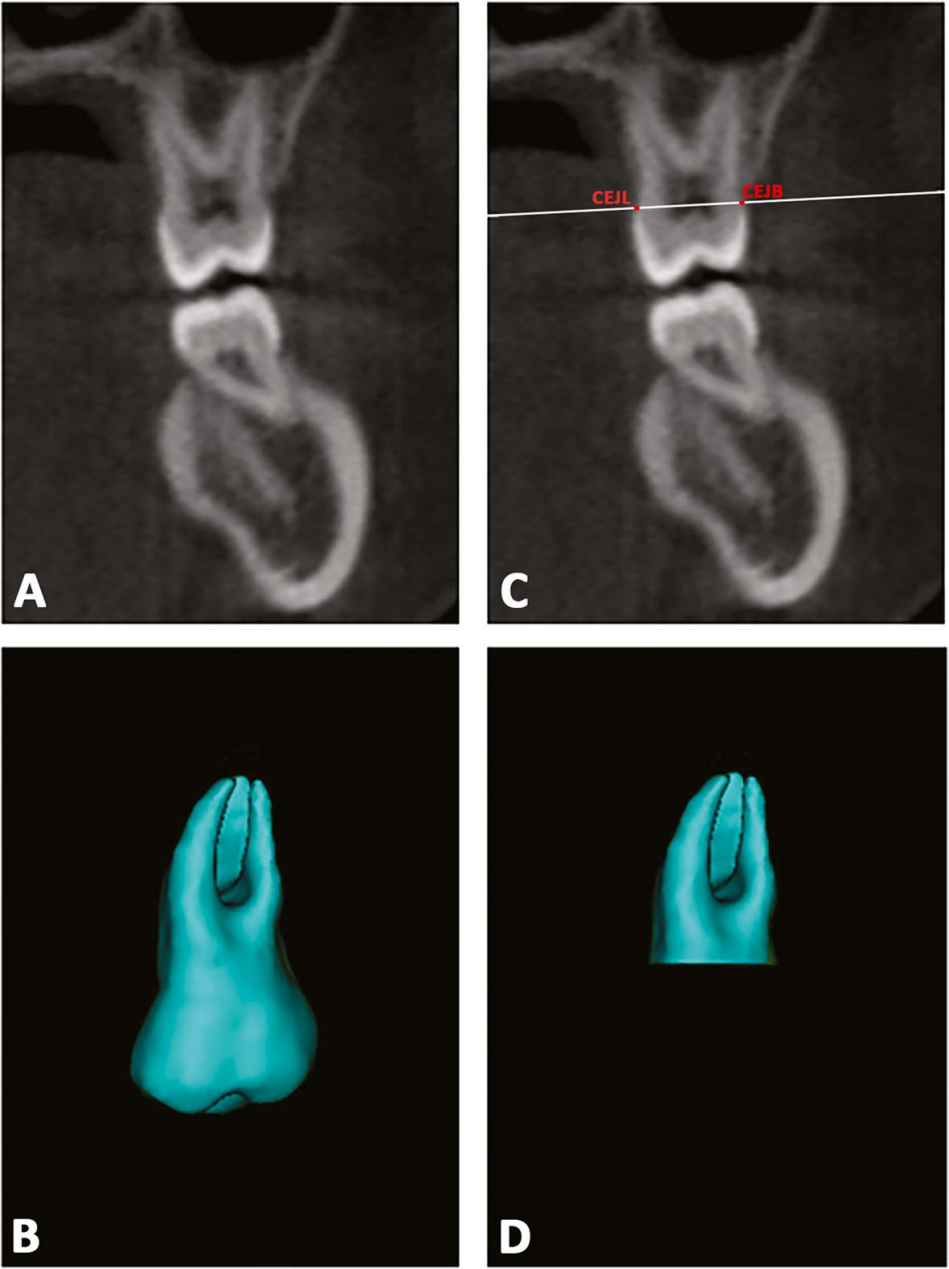
**a**, **b** T0 mask of each tooth was duplicated, and a second model was obtained. **c**; definition of the plane cut passing through two landmarks placed on the midpoint of palatal and buccal aspects of the crown at the cementoenamel junction (CEJ) level; (**d**) generation of the radicular 3D model template for each tooth investigated

In the Mimics software, each segmented tooth at T0 was duplicated (Fig. 4a, b) and two landmarks were located on the lingual (CEJL) and buccal (CEJB) aspects of the crown at the cementoenamel junction level, on both original and duplicated models. A specific plane passing through these points was drawn and the duplicated model was cut along this plane to reproduce the radicular 3D model for each tooth (Fig. 4c, d). Finally, the final radicular templates were imported on 3-Matic Medical software.

### Step 4 – First superimpositions (T0, T1 3D models) and surface-based registration

**Fig. 5:**
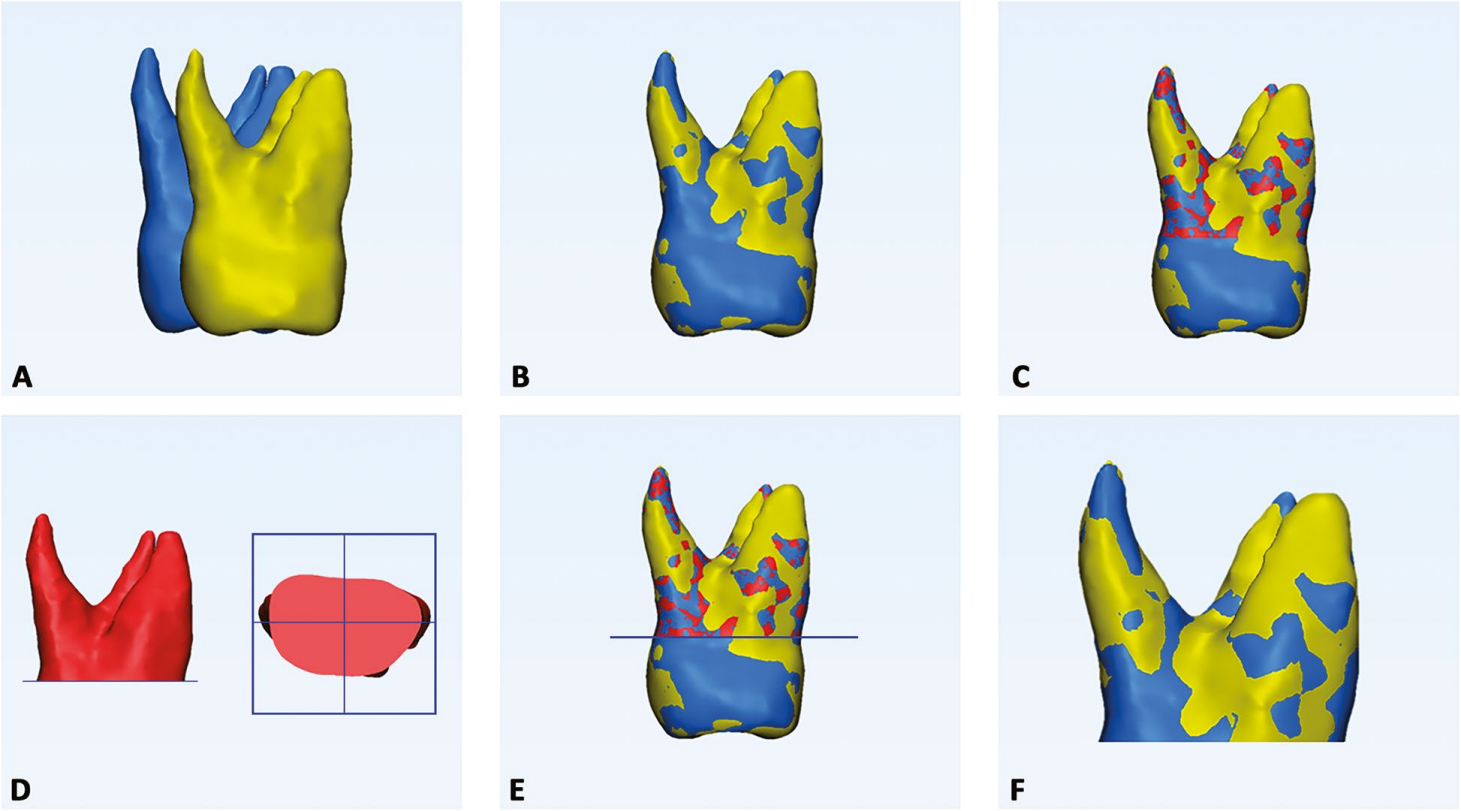
Registration of T0-T1 3D tooth models, and crown removal from 3D models. **a** Preliminary point-based superimposition, by selecting five random points on the buccal, palatal/lingual, mesial approximal, distal approximal, and occlusal aspects of the two models of the same tooth; (**b**) global registration using best-fit algorithm; (**c**) T0 tooth model (light blue) and radicular template (red) and T1 tooth model (yellow) in the same spatial orientation after superimposition; (**d**,**e**) definition of a single plane cut by randomly selecting three points on the lower surface of the T0 radicular template; (**f**) generation of the final T0 and T1 radicular models

A point-based superimposition between T0 and T1 original models was carried out by landmarking five random points on the buccal, palatal/lingual, mesial approximal, distal approximal, and occlusal aspects of 3D models [13]. Then, a global surface-based registration (best fit) of the 3D tooth models was obtained (Fig. 5a, b). The 3-Matic Medical software was used for this purpose.

### Step 5 – Crown cut from 3D models

Since both T0 and T1 original models and the radicular template had the same spatial orientation obtained in Step 4 (Fig. 5c), it was possible to remove the crown from the teeth at the same level. For this purpose, three points were randomly selected on the lower surface of the T0-3D radicular model (generated in step 3) to create the plane cut. Thus, the final T0 and T1 radicular models were generated for each tooth investigated (Fig. 5d-f).

### Step 6 – Volume measurement, 3D Deviation analysis, and matching percentage calculation

**Fig. 6:**
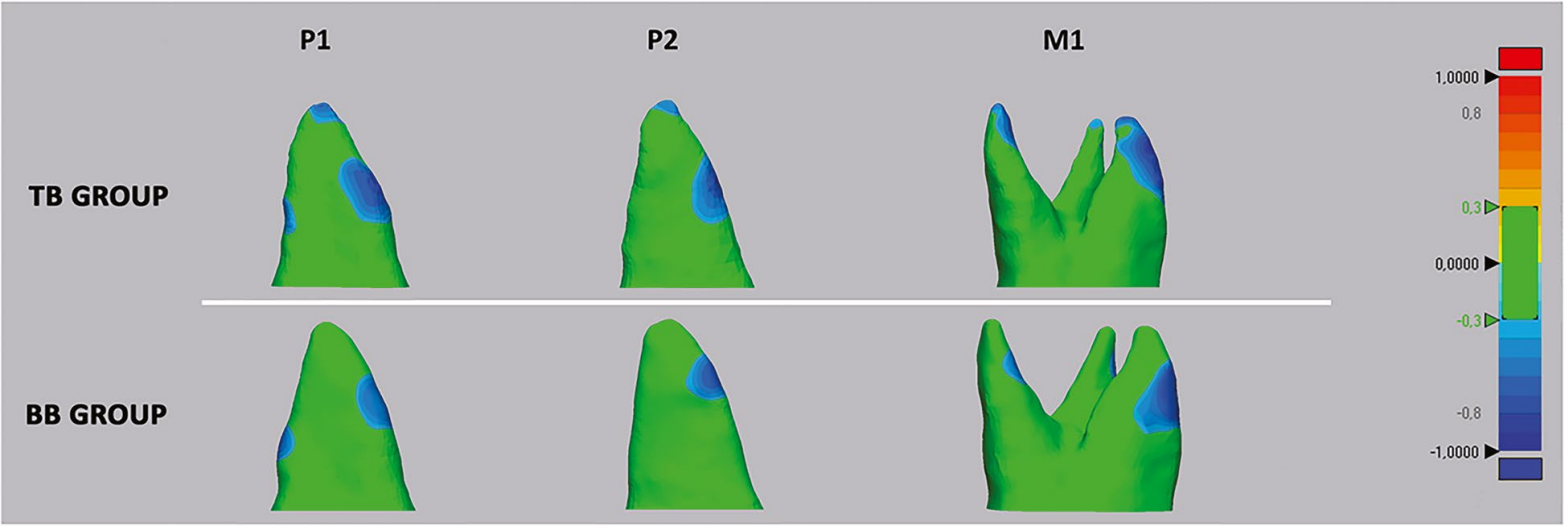
Deviation analysis between the T0 and T1 radicular models of first molar (M1), first premolar (P1) and second premolar (P2) in both tooth-borne (TB) and bone-borne (BB) expander groups. The colored map shows the deviations (negative blue, positive red) between the mesh models. The range of tolerance (green colour) was set a ± 0.3 mm. The colour-coded map showed that the reduction of cementum (blue-tone) was localized in the apical, bucco-apical and bucco-medial radicular areas of both abutment and un-anchored teeth in the TB group. A similar resorption pattern was identified in the BB group, despite the absence of detectable deviation at the apex

The radicular 3D models were imported into Geomagic Control X software (version 2017.0.0, 3D Systems, CA, USA). At first, the root volume was measured along with the percentage of the radicular volume loss between T0 and T1 (primary outcomes). Then, surface-based deviation analysis was carried out to calculate the Euclidean distance between the two superimposed 3D radicular models based on data from all points of the surface shells (secondary outcomes). The values were represented on a color map which showed surface deviations according to the range of tolerance (± 0.3 mm) (Fig. 6). The percentages (%) of all the distance values within the tolerance range were also calculated; these values represented the degree of matching between the pairs of root 3D models and reflected the surface changes after RME.

Using Mimics Medical Software, the amount of skeletal maxillary expansion (PW = palatal width) and dento-alveolar expansion (DAW = dento-alveolar width) was calculated at level of P1, P2 and M1 according to consolidated methodology [20]. These data would serve for assessing the correlation between the amount of maxillary expansion (skeletal and dentoalveolar) and ERR.

The digital workflow and related measurements were performed by a single examiner, with 10 years of orthodontic research experience on CT scans of craniofacial bones. The examiner processed only 3 CBCT scans each day to avoid fatigue. Ten patients were randomly selected, and the entire procedure was repeated by the same expert investigator after four weeks. The same patients were also re-measured by a second expert operator. Volumetric and linear changes of the upper P1, P2 and M1 and linear changes of PW and DAW were used to assess the reliability of the methodology.

### Statistical analysis

A preliminary evaluation of sample size power was performed on 20 subjects (10 in the TB group and 10 in the BB group). The analysis suggested that 15 patients were required in each group to reach the 80% power to detect a mean difference of 3,17 mm3 between the radicular volumetric changes recorded between P1 and M1 in the BB group, with a confidence level of 95% and a beta error level of 20%. However, according to the inclusion criteria, we were able to include 20 subjects in each group which increased the robustness of the data. The normal distribution and equality of variance of the data were performed with Shapiro–Wilk Normality Test and Levene’s test. Since the data showed homogeneous variance, parametric tests were used to evaluate and compare measurements.

The chi-square test and Student’s t-test were used respectively to assess the homogeneous distribution of gender and age variables between TB and BB groups. Means, standard deviations, and minimum and maximum values for root length and volume were calculated for each tooth in both treated and control groups at each time point. The one-way analysis of variance (ANOVA) was used to evaluate the changes of radicular volumes, radicular lengths, and percentage matching among the investigated teeth; in case of statistical significance, the Bonferroni test was performed for post hoc comparisons. The unpaired Student’s t-test was used to investigate the changes in radicular volume, radicular length, percentage of matching (T0-T1 superimpositions) and maxillary widths (PW and DAW) between TB and BB groups, for each tooth investigated. The same test was also used to perform a preliminary comparison between the right and left side; since no differences were found, right and left teeth of the same type were pooled [13]. Multiple linear regression was used to evaluate the influence of the expander type and skeletal and dentoalveolar expansion (predictors) on the amount of ERR (dependent variable).

Intra-examiner and inter-examiners reliability was assessed using the intraclass correlation coefficient (ICC). Data sets were analyzed using SPSS® version 24 Statistics software (IBM Corporation, 1 New Orchard Road, Armonk, New York, USA).

## Results

The demographic characteristics of the study sample are reported in Table 1. No differences were found between TB and BB groups concerning gender distribution. However, differences were detected between the two groups according to age distribution; subjects in the TB were about 1.5 years younger than those included in the BB group, which was consistent with the clinical objectives established, according to the potentially different maturational stage of the mid-palatal suture [1].

**Table 1:** Demographic characteristics of the study sample

Sample characteristics	Total Sample (*n* = 40)	TB group	BB group	Significance
		**(*n* = 20)**	**(*n* = 20)**	
**Gender: male/female**	17/23	9/11	8/12	NS*
**Age, y: mean (SD)**	13,8 (1,33)	13,1 (1,08)	14,5 (1,1)	*p* < 0.05**

*NS* Not significant

^*^P value set as ≤ 0.05. and assessed chi-square test

^**^P value set as ≤ 0.05. and assessed by Student's t test

In both TB and BB groups, a significant volumetric loss was found among the investigated teeth (*p* < 0.05), although to a different extent (Table 2). M1 showed greater volumetric changes compared to P1 and P2 (*p* < 0.05) in both groups. The volumetric loss was significantly and remarkably greater in the TB group compared to the BB group for each tooth investigated (*p* < 0.05). However, when the radicular volumetric loss was calculated as a percentage of the total radicular volume, no differences were found among the investigated teeth in each group. Also, the percentage of volumetric loss was significantly greater in the TB group (*p* < 0.05). No differences were found in the control sample (lower teeth) for all the parameters investigated between T0 and T1 (*p* > 0.05).

**Table 2:** Descriptive and inferential statistics of radicular volumetric changes (mm3 and percentage) occurred after maxillary expansion

	**T0-T1 (mm3)**									**T0-T1 (%)**								
			**TB Group**			**BB Group**						**TB Group**			**BB Group**			
**Upper (Test)**	***Teeth***	***n***	***Mean***	***SD***	p value*	***Mean***	***SD***	p value*	p value**	***Teeth***	***n***	***Mean***	***SD***	p value*	***Mean***	***SD***	p value*	p value**
	**P1**	20	9,74 (c)	3,77	*p* < 0.05	1,45 (c)	0,64	*p* < 0.05	*p* < 0.05	**P1**	20	8,11	2,85	NS	1,23	0,7	NS	*p* < 0.05
	**P2**	20	8,23 (c)	3,11		1,68 (c)	0,53		*p* < 0.05	**P2**	20	6,35	2,25		1,41	0,52		*p* < 0.05
	**M1**	20	26,21 (a,b)	10,03		4,62 (a,b)	3,12		*p* < 0.05	**M1**	20	8,31	3,01		1,47	1,03		*p* < 0.05
**Lower (Control)**	***Teeth***	***n***	***Mean***	***SD***	p value*	***Mean***	***SD***	p value*	p value**	***Teeth***	***n***	***Mean***	***SD***		***Mean***	***SD***	p value*	p value**
	**P1**	20	-0,25	0,94	NS	-0,12	0,76	NS	NS	**P1**	20	-0,16	0,75	NS	-0,12	0,59	NS	NS
	**P2**	20	-0,06	0,76		0,05	0,41		NS	**P2**	20	-0,05	0,59		0,04	0,5		NS
	**M1**	20	0,37	1,34		0,26	1,21		NS	**M1**	20	0,11	0,43		0,08	0,81		NS

*P1* First premolar, *P2* Second premolar, *M1* First molar, *TB* Tooth borne, *BB* Bone borne, *T0* Pre-treatment, *T1* Post-retention, *n* Number of teeth, *SD* Standard deviation, *NS* Not significant

*p* value* based on one-way analysis of variance (ANOVA) for intra-group comparison (different teeth) and set at *p* < 0.05; post-hoc assessment performed according to the Bonferroni's multiple comparisons test

*p* value** based on Independent Student's t test for inter-groups comparison and set at *p* < 0.05

In both TB and BB groups, all the investigated teeth showed a significant reduction of the radicular length from T0 to T1 (*p* < 0.05), although to a different extent (Table 3). M1p was the root mostly affected by length reduction (*p* < 0.05). The changes in radicular length were significantly and remarkably greater in the TB group compared to the BB group for each tooth investigated (*p* < 0.05). No differences were found in the control sample (lower teeth) for all the parameters investigated (*p* > 0.05).

**Table 3:** Descriptive and inferential statistics of radicular lenght changes occurred after maxillary expansion

	**T0-T1 (mm)**										**T0-T1 (mm)**								
			**TB Group**			**BB Group**							**TB Group**			**BB Group**			
	***Teeth***	***n***	***Mean***	***SD***	p value*	***Mean***	***SD***	p value*	p value**				***Mean***	***SD***	p value*	***Mean***	***SD***	p value*	p value**
**Test**	**P1**	20	0,51 (c)	0,18	*p* < 0.05	0,08 (b,e)	0,03	*p* < 0.05	*p* < 0.05	**Control**	**P1**	20	0,02	0,21	NS	-0,05	0,30	NS	NS
	**P2**	20	0,39 (e)	0,14		0,11 (a,e)	0,02		*p* < 0.05		**P2**	20	-0,06	0,13		0,04	0,11		NS
	**M1m (c)**	20	0,37 (a,e)	0,10		0,10 (e)	0,04		*p* < 0.05		**M1m (c)**	20	0,09	0,48		0,03	0,35		NS
	**M1d (d)**	20	0,43	0,14		0,09 (e)	0,02		*p* < 0.05		**M1d (d)**	20	0,01	0,52		0,04	0,55		NS
	**M1p (e)**	20	0,56 (b,c)	0,19		0,15 (a,b,c,d)	0,04		*p* < 0.05		-	-	-	-		-	-	-	

*P1* First premolar, *P2* Second premolar, *M1m* First molar mesial root, *M1d* First molar distal root, *M1p* First molar palatal root, *TB* Tooth borne, *BB* Bone borne, *T0* pre-treatment, *T1* post-retention, *n* Number of teeth, *SD* Standard deviation

*p* value* based on one-way analysis of variance (ANOVA) for intra-group comparison (different teeth) and set at *p* < 0.05; post-hoc assessment performed according to the Bonferroni's multiple comparisons test

*p* value** base on Independent Student's t test for inter-groups comparisons and set at *p* < 0.05

In both TB and BB groups, significant differences in the percentage of matching were found among P1, P2, and M1 (*p* < 0.05) when superimposing T0 to T1 3D models (Table 4). The M1 showed a limited percentage of matching compared to P1 and P2. All the investigated teeth showed a significantly higher percentage of matching in the TB group for T0-T1 shells superimposition (*p* < 0.05).

**Table 4:** Comparison of matching percentage of pre-treatment and post-retention radicular shells (T0-T1 superimposition) for each tooth investigated

		**T0-T1 matching (%)**						
		**TB Group**			**BB Group**			
**Teeth**	**n**	***Mean***	***SD***	*p* value*	***Mean***	***SD***	*p* value*	*p* value**
**P1 (a)**	20	83,89 (c)	3,73	*p* < 0.05	92,90 (c)	3,20	*p* < 0.05	*p* < 0.05
**P2 (b)**	20	86,03 (c)	4,18		94 (c)	2,90		*p* < 0.05
**M1 (c)**	20	77,16 (a,b)	5,27		86,77 (a,b)	4,18		*p* < 0.05

*P1* First premolar, *P2* Second premolar, *M1* First molar, *n* Number of teeth, *SD* Standard deviation

*p* value* based on one-way analysis of Variance (ANOVA) for intra-group comparisons (different teeth), and set at *p* < 0.05

*p* value** for inter-groups comparisons, based on Independent Student's t test and set at *p* < 0.05

Concerning the effectiveness of both appliances in expanding the maxillary arch, the PW changes (skeletal expansion) were consistently greater in the BB group compared to the TB group, for each level of measurements (P1, P2, M1) (*p* < 0.05). At the same time, DAW changes (dentoalveolar expansion) were significantly greater in the BB group only for M1 measurements (Table 5).

**Table 5:** Skeletal maxillary expansion (PWE = palatal width expansion) and dento-alveolar expansion (DAE = dento-alveolar expansion) recorded between T0-T1 (negative values) in both tooth-borne (TB) and bone-borne (BB) group

	**T0-T1**						
			**TB Group**		**BB Group**		***p* value**
		**n**	***Mean***	***SD***	***Mean***	***SD***	
P1	**PWE**	20	-1,83	0,64	-2,62	1,22	*p* < 0.05
	**DAE**	20	-5,04	1,79	-4,14	1,92	NS
P2	**PWE**	20	-1,60	0,51	-2,29	1,15	*p* < 0.05
	**DAE**	20	-4,73	1,81	3,99	1,50	NS
M1	**PWE**	20	-1,54	0,48	-2,22	1,13	*p* < 0.05
	**DAE**	20	-5,59	1,68	-4,38	1,57	*p* < 0.05

*p* value based on Independent Student's t test for inter-groups comparisons and set at *p* < 0.05

According to multiple linear regression analysis, there was, in general, a moderated significant correlation between ERR and predictors with a stronger influence for the type of expander compared to both skeletal and dentoalveolar expansion (*p* < 0.05) (Table 6).

**Table 6:** Multiple linear regression analysis using root resorption as dependent variable and expander type (EXPANDER), palatal expansion (PWE) and dento-alveolar expansion (DAE) as predictive variables

	**Model**		**Unstandardized coefficients**		**Standardized coefficients**	**t**	***p* value**	**95,0% Interval Coefficient**	
		**R-squared**	**B**	**Standard Error**	**Beta**			**Lower Limit**	**Upper Limit**
P1	(Costant)	0,760	14,66	1,84	-	7,96	0,00	10,92	18,39
	EXPANDER		-8,02	0,96	-0,82	-8,37	0,00	-9,96	-6,08
	PWE		-0,26	0,48	-0,05	-0,54	0,59	-1,23	0,71
	DAE		-0,52	0,25	-0,20	-2,08	0,04	-1,03	-0,01
P2	(Costant)	0,746	17,29	1,53	-	11,31	0,00	14,19	20,39
	EXPANDER		-6,41	0,76	-0,82	-8,44	0,00	-7,95	-4,87
	PWE		0,58	0,41	0,14	1,43	0,16	-0,24	1,41
	DAE		0,36	0,22	0,15	1,67	0,10	-0,08	0,81
M1	(Costant)	0,771	34,97	5,80		6,03	0,00	23,21	46,74
	EXPANDER		-20,79	2,72	-0,80	-7,65	0,00	-26,30	-15,28
	PWE		-1,78	1,50	-0,12	-1,19	0,24	-4,82	1,25
	DAE		-1,66	0,79	-0,22	-2,09	0,04	-3,27	-0,05

*p* values set at *p* < 0.05

Concerning the reliability of the methodology, no differences were found between intra-operator readings, with excellent correlation indexes ranging from 0,913 to 0,941 for radicular volumes assessments and ranging from 0,901 to 0,923 for linear measurements. Also, no differences were found between intra-operator readings, with correlation indexes ranging from 0,874 to 0,899 for radicular volume assessments and ranging from 0,883 to 0,912 for linear measurements.

## Discussion

Previous studies reported that RME with a tooth-borne expander could determine root resorption since heavy forces are transmitted to the maxilla by abutment teeth [2]. In order to reduce burden and adverse effects on the dentition, a maxillary expander supported completely or partly by skeletal anchorage devices was proposed [1]. Nevertheless, there is limited comparative evidence between RME assisted by dental and skeletal anchorage and ERR. In particular, one study [9] tested asymmetric anchorage systems in the same appliance (tooth-tissue-borne on one side and bone-borne on the other side); instead, another study [21] did not include pure bone-borne anchorage systems in the investigation. To the best of our knowledge, this is the first study in the literature that investigates three-dimensionally the post-expansion radicular changes of posterior maxillary teeth in patients treated either with tooth-borne or bone-borne rapid maxillary expansion appliances.

In the present investigation, rapid maxillary expansion (RME) led to a reduction in radicular (root) volume in the maxillary first molars and first and second premolars. The extent of volumetric loss was more pronounced when using the TB (tooth-borne) expander. Notably, the first molars exhibited a greater volumetric reduction compared to the other investigated teeth. However, when the volumetric loss was expressed as a percentage of the total radicular volume, no statistically significant differences were observed among the examined teeth. This finding suggests that posterior teeth, whether functioning as abutment teeth (specifically, P1 and M1 in this study) or un-anchored teeth (like P2), may be equally susceptible to ERR despite differences in the load they experienced, as also suggested by previous evidence [7, 8, 11]. However, these data contrast with another study that showed no resorption on non-banded premolars, suggesting that these teeth moved laterally with the alveolar process [22]. Further studies are warmly encouraged to better elucidate this aspect, even concerning different appliance designs.

The volumetric loss detected in the TB group was similar to that reported by a previous study testing a conventional maxillary expander [15]. Considering that the age of the study sample (TB = 13,1 ± 1,08; BB = 14,5 ± 1,11) approximates nearly the final maturational stage of the premolars [23], it could be assumed that the ERR detected may have disrupted the final developmental stage of these teeth, however with a remarkable less extent in the BB group.

We also assessed radicular length changes and the deviation analysis between the radicular 3D models, superimposed at different time points, to clarify the pattern of ERR involved during RME. All investigated teeth reported a reduction of radicular length: the P1 and M1p were the roots mostly affected by length reduction in the TB group (respectively 0,51 mm and 0,56 mm of length reduction). At the same time, M1p was the root mostly affected by length reduction in the BB group (0,15 mm of length reduction). Although the main concerns of root resorption is the harmful consequence of root shortening on tooth longevity, the values recorded in this study should be far from threatening the function of the dentition since 2 mm of root shortening was found to reduce the total attachment area of 5–10% [24, 25].

According to the color-coded map, the reduction of cementum (showed by blue-tone) was localized in the apical, bucco-apical and bucco-medial radicular areas of posterior dentition in the TB group. A similar pattern of resorption was identified in the BB group, although the modifications in this region were almost irrelevant, as confirmed by the linear measurements of radicular length. These findings corroborate previous evidence from histological materials showing the generation of radicular resorption on the buccal surface of the roots in the form of small irregularly shaped lacunae [6, 8] and also from a recent well-conducted micro-CT studies [9]. This pattern of ERR occurs since the forces generated by RME are orientated toward the buccal side of dentoalveolar arch, causing the compression of the periodontal ligament and subsequent hyalinization on the buccal side of the roots. ERR occurs during the elimination of the hyalinization tissue on the compressed side [26]. Moreover, the root apex may exhibit heightened responsiveness due to the amplification of force per unit surface area that occurs in this region during rapid maxillary expansion (RME). This assumption can be explained by the presence of a thicker and more rigid bone in this area compared to the trabecular bony architecture of the cervical region [27].

The null hypothesis in the present study was rejected since ERR was significantly more extended in the TB (tooth-borne) group compared to the BB (bone-borne) group. This finding is likely explained by the absence of direct forces exerted on the dentition in the BB group, which is consistent with the outcomes of a recent split-mouth study [9]. However, it is important to note that a direct comparison between the two studies is not entirely appropriate, as the authors of the previous study evaluated a maxillary expander with skeletal anchorage on one side and tooth-tissue-borne anchorage on the other side. This appliance design may have introduced an asymmetric distribution of forces, potentially subjecting the teeth on the tooth-tissue anchored side to higher loads. Instead, our findings corroborate those from another study [28] although the authors have restricted the observation to the analysis of radicular length (2D analysis).

It could be argued that the amount of ERR is primarily influenced by the amount of expansion rather than by the appliance design. In this regard, we calculated the amount of skeletal and dentoalveolar expansion and we used multiple linear regression to assess the influence of these variables, compared to the type of expander, on the ERR recorded. ERR showed moderate correlation with the independent variables, but with higher predictive values for the type of expander used. However, the limited sample size and the restricted range of PW and DAW changes recorded could have contributed to increase the weight of categorical variable (type of expander) over the maxillary expansion in the correlation with ERR.

Although the present findings suggest that BB-RME induce less root resorption compared to TB-RME, the magnitude of the differences could be considered clinically questionable. In this regard, the usage of skeletal anchorage for supporting RME should be based on other specific factors such as the patient’s age, skeletal maturation, baseline dentoalveolar compensation of transverse maxillary deficiency, and tooth eruption status. The amount of volume loss and length reduction (minimal) found in the BB group may be related to the design of the skeletal anchorage system, which consists of two mini-screws placed on the palatal slope between the second premolar and the first molar area. This area of placement of miniscrews may have generated force vectors that may have been minimally transmitted to the dentition. In this regard, further studies testing different skeletal anchorage designs and using a consistent methodology for evaluating ERR are warmly recommended to provide conclusive evidence.

Lastly, subjects enrolled in the present study were adolescents with an advanced maturational stage of the midpalatal suture compared to pre-pubertal stage [29]. Thus, the amount of ERR detected in the posterior dentition could have been influenced by higher skeletal resistances during the expansion procedure.

## Limitations

1. The absence of a control group of untreated subjects is certainly the main limitation of the present study, although we avoided unnecessary radiation exposure according to the A.L.A.D.A. principle [30]. We used the lower dentition as the control group; however, it could be argued that lower teeth are not free from induced movement during RME, especially in the presence of a cross-bite relationship that increases occlusal interferences and the forces transmitted [31]. This could explain the minimal reduction of volume and length of the first molar roots since they were the teeth mostly involved in the cross-bite relationship. Conversely, we found a very small volumetric and linear augmentation in the premolar region. Considering the age range of the included patients, it is difficult to understand whether these changes depend on root development or the continuous cementum formation due to changes in the occlusion [13].
2. We used CBCT scans with voxel size of 0.3 mm, however there are contrasting indications on the adequate spatial resolution of CBCT examinations for assessing radicular volume. In this regard, a previous study suggested that ERR could be underestimated with voxel sizes greater than 0.2 mm, [32] while recent evidences suggested no significant differences in sensitivity and specificity between 0.3 mm voxel size (used in the present study) and 0.15, 0.20, 0.25 mm voxel sizes, but with the advantage of lower radiation exposure [33].

## Conclusion

- A significant greater amount of root resorption was observed with TB-RME compared to BB-RME, at post-expansion stage.
- The ERR was located in the apical, bucco-apical and bucco-medial radicular areas of 3D radiclar models. Even non-anchored teeth were affected by ERR, suggesting that the transmission of the forces is not limited to the abutment dentition.

## Data Availability

The datasets used and analysed during the current study are available from the corresponding author on reasonable request.

## Abbreviations

ERR: External root resorption
TB: Tooth-borne
BB: Bone-borne
RME: Rapid maxillary expansion
CBCT: Cone-beam computed tomography
M1: Maxillary first molars
M1m: Maxillary first molars mesial root
M1d: Maxillary first molars distal root
M1p: Maxillary first molars palatal root
P2: Second premolars
P1: First premolar
3D: Three-dimensional
SEM: Scanning electron microscope
micro-CT: Micro-tomography
FOV: Field of view
DICOM: Digital Imaging and Communications in Medicine
ROI: Region of Interest
CEJL: Lingual aspects of the crown at the cementoenamel junction level
CEJB: Buccal aspects of the crown at the cementoenamel junction level
PW: Palatal width
DAW: Dento-alveolar expansion
ANOVA: One-way analysis of variance
ICC: Intraclass correlation coefficient
A.L.A.D.A: As low as low as diagnostically acceptable
